# Workers compensation-reported injuries among security and law enforcement personnel in the private versus public sectors

**DOI:** 10.1186/s40621-018-0156-9

**Published:** 2018-07-02

**Authors:** W. S. Witt, T. L. Bunn, S. Slavova

**Affiliations:** 1 Kentucky Injury Prevention and Research Center, University of Kentucky, Lexington, KY USA; 2 Department of Epidemiology, University of Kentucky College of Public Health, Lexington, KY USA; 3 Department of Preventive Medicine and Environmental Health, University of Kentucky College of Public Health, Lexington, KY USA; 4 Department of Biostatistics, University of Kentucky College of Public Health, Lexington, KY USA

**Keywords:** Private and public security and law enforcement sectors, Fall injuries, Strain injuries, Job tenure, First reports of injuries

## Abstract

**Background:**

Private and Public security and law enforcement (SLE) sectors perform multiple overlapping job duties.

**Methods:**

Workers’ compensation (WC) SLE first reports of injury (FROI) data (2005–2015) were analyzed to describe injuries, identify differences in awarded WC benefits, and compare the probability of a FROI resulting in awarded benefits between Public and Private SLE. A Pearson’s chi-square test was utilized and reverse selection logistic regression was performed to estimate the odds ratio that a FROI would result in an awarded benefit for Private vs. Public SLE, while adjusting for relevant covariates.

**Results:**

Private SLE had higher FROI percentages for younger and for older workers, fall injuries, and back injuries, compared to Public SLE. The adjusted odds that a FROI resulted in an awarded benefit was 1.4 times higher for Private SLE compared to Public SLE; (95% confidence interval [CI] = 1.09,1.69). Middle-aged SLE employee adjusted odds of awarded benefits was 3.3 times (95% CI [1.96, 5.39]) higher compared to younger employees. Adjusted odds of awarded benefits was 3.8 times (95% CI [1.34, 10.61]) higher for gunshots and 1.7 times (95% CI [1.22, 2.39]) higher for fractures/dislocations compared to other nature of injuries. Motor vehicle injury, fall/slip, and strain related FROIs had elevated adjusted odds of awarded benefits compared to other injury causes.

**Conclusions:**

Results highlight the importance of injury prevention education and worker safety training for Private and Public SLE sector workers on fall prevention (especially in Private SLE) and strain prevention (especially in Public SLE), as well as motor vehicle safety.

## Background

Security and law enforcement (SLE) in the United States crosses multiple industries, with a robust presence in both the Public and Private sectors. There are approximately 18,000 Public sector law enforcement agencies in the U.S., ranging from local police departments with 10 or fewer officers, to large municipal and state forces (Banks et al. 2016). While estimates vary, nationwide employment for Public law enforcement personnel in 2012 was approximately 750,000 sworn and 325,000 non-sworn employees. State and local police forces constitute the majority of Public sector law enforcement, and are composed of sworn officers and non-sworn civilian employees (Banks et al. 2016). Sworn officers possess full arresting powers, and are permitted to carry firearms and badges. Non-sworn employees provide support to primary law enforcement functions. There were 7200 police officers and 1600 police supervisors employed in 2016 (Bureau of Labor Statistics 2017c). In Kentucky, employment numbered 7870 for Public service police and detectives, and 1600 for police and detective supervisors (Bureau of Labor Statistics 2017a).

Counterparts in the Private sector are defined under broader terms. The largest Private security association in the U.S., ASIS International, defines Private security as the “nongovernmental, Private-sector practice of protecting people, property, and information, conducting investigations, and otherwise safeguarding an organization’s assets… [which] may be performed for an organization by an internal department or by an external, hired firm” (ASIS International 2009). The occupations included in the Private sector, as defined through the federal government’s Standard Occupation Classification (SOC) system, are detectives, guards, armored car services, and security systems services (Strom et al. 2010). Compared to Public SLE, there are more personnel in the Private sector. According to the BLS, in year 2016, there were approximately 1.1 million security guards, and 29,000 private detectives and investigators employed in the United States (Bureau of Labor Statistics 2017a, b), of which, 11,050 security guards, and 140 private detectives were employed in Kentucky (Bureau of Labor Statistics 2017c).

The overall U.S. law enforcement fatal injury rate was estimated to be 16.4 fatalities per 100,000 workers (Tiesman et al. 2013). In 2016, police officers and detectives experienced injury rates above the national average for all occupations, while security guards experienced an injury rate below the national average within the Private industry sector (Bureau of Labor Statistics 2017d). In the Public sector, police officers had a rate of 498.3 nonfatal injuries and illnesses involving days away from work per 10,000 full-time workers, while Public sector detectives and criminal investigators had injury rates of 284.6 nonfatal injuries per 10,000 full-time workers.

In the United States, workers’ compensation is a form of occupational injury insurance that reimburses workers for expenses such as lost wages and medical treatment, in the event of an injury acquired on the job. Data collected through workers’ compensation can be a useful tool for occupational injury surveillance (Holloway-Beth et al. 2016; Bunn et al. 2011). Holloway-Beth et al. (2016) found a disproportionately high number of claims among correctional officers, relative to their proportion of the state’s law enforcement employment. Bunn et al. (2011) found that Private sector solid waste collectors had greater odds of compensated first reports of injuries (FROIs) compared to those in the Public sector. Workers’ compensation data has also been used to explore urban-rural differences in work disability duration (Young et al. 2008; Fan et al. 2013).

Public and Private police officers overlap and collaborate in multiple job activities. Public police functions are carried out in the Private sector, Public police cooperate on a daily basis with Private police in private and public areas, and Private police carry out investigations that inform Public police efforts (Sparrow 2014). Differences in injury compensation between Public and Private SLE workers is unknown at both the national and state levels, and workers’ compensation data is a data source that can be used to supplement BLS survey data on injuries in the Public vs. Private SLE. The objectives of this study were to (1) describe injuries among Public SLE vs. Private SLE; (2) identify differences in awarded workers’ compensation benefits associated with Public vs. Private SLE FROIs; and (3) compare the probability of a FROI resulting in awarded benefits between Public and Private SLE.

## Methods

### Study population and selection criteria

De-identified Kentucky workers’ compensation FROIs for years 2005–2015 were obtained from the Kentucky Department of Workers’ Claims. All Kentucky employers with one or more employees are required to have workers’ compensation coverage; certain worker populations are exempt such as agricultural workers, but they may elect coverage. Kentucky Revised Statute 342.038 requires FROIs be reported by the employer to its workers’ compensation insurance carrier within three working days of incident notification, and by the employer’s insurance carrier to the state Department of Workers’ Claims within one week of receiving notification. “Claims” are only cases in which there are disagreements that cannot be resolved. Claims are categorized by county of residence of the Plaintiff, and assigned to an Administrative Law Judge (ALJ). The claim is then scheduled for a Benefit Review Conference, at one of 10 Hearing Sites across Kentucky. One of the ALJs is appointed the Chief ALJ who supervises the other ALJs. According to the Kentucky Department of Workers’ Claims, the following are inclusion criteria for FROIs (Kentucky Regulatory Statute 1980): All worker injuries that require at least one day off from work or result in a disability that extends beyond 60 days are required to be reported. Further inclusion criteria for FROIs in this study were for all accepted cases; open or closed FROIs; FROIs for claimants of all ages and those with unknown ages; FROIs for out-of-state residents who were injured in Kentucky, as well as for Kentucky residents who were injured out-of-state; and ‘Lost-time’ FROIs. Reimbursement for medical expenses related to injuries was not available in the FROI data set.

To be eligible for workers’ compensation benefits (indemnity and/or lump sum payments), a worker needs at least 7 days lost work time due to an injury or has to have a permanent partial disability with no missed workdays due to an injury. Indemnity payments associated with FROIs or claims are defined as paid income benefits to compensate for lost wages, functional impairment, or death. When a worker has lost at least 2 weeks of work time due to an injury, the worker is eligible for lost wage compensation retroactive to the first day of work lost.

### Study design

This study incorporated a cross-sectional design. Selection of SLE FROIs was based on North American Industry Classification System (NAICS) codes, and Standard Occupational Classification (SOC) system codes; in cases where SOC codes were missing or improperly coded, the occupation text field was reviewed to identify occupation. A key word narrative text search for ‘police’ within the occupation field was used to increase identification of relevant cases, and similar occupations were grouped together. Security guards (SOC code: 33–9032) and Private detectives and investigators (SOC code: 33–9021) were grouped into “guards and police, except Public service;” police & sheriff’s patrol officers (SOC code: 33–3051) and detectives and criminal investigators (SOC code: 33–3020) were grouped into “police and detectives, Public service”. First-line supervisors of law enforcement workers (SOC code: 33–1010) were grouped into “supervisors of police and detectives;” and all remaining occupations were represented by “all other.”

The final dataset contained 4377 records averaging approximately 400 records per year, with 3478 in the Public sector and 899 in the Private sector. Industry sector (Public vs. Private) was determined using NAICS codes. The University of Kentucky institutional review board approved the study (IRB number 05–0231-P2J). The study involved analysis of secondary data with no personal identifiers, so no informed consent was needed or obtained.

#### Measures

Reported SLE worker injuries were described by gender, age group, nature of injury, cause of injury, injured body part, season of injury, geographical region where the injury occurred, worker’s residence region, occupation, industry, and length of time off work after the injury. Causes of injury were grouped into the following categories: absorption, ingestion or inhalation; cut, puncture, or scrape; fall/slip; motor vehicle-related; person in act of crime; strain; struck by animal or object; struck by fellow worker, patient or other person; and all other causes.

One outcome of interest was if the FROI resulted in an awarded benefit (yes or no). A FROI resulted in an awarded benefit if the disposition status indicated 1) agreement approved – ALJ; 2) award – ALJ; 3) lump sum agreement on first report; and 4) agreement approved on first report. The “Other” disposition category included 1) appealed to Supreme Court; 2) administratively closed; 3) assigned to ALJ; 4) Case dismissed-ALJ; 5) Consolidated ALJ award; 6) held in abeyance; and 7) medical dispute concluded.

### Statistical analyses

Descriptive analysis and Pearson’s chi-square test was utilized to evaluate demographic and injury characteristic differences between the SLE sectors. Simple logistic regression was used to initially assess the association between the outcome of interest (a FROI resulting in an awarded benefit), and the main exposure of interest (SLE sector). Multiple factors were considered as potential covariates and included in a full logistic regression model, including gender, age group, length of time on the job, season of injury, worker residence (Appalachia vs. non-Appalachia), worker geographic location of injury (Appalachia vs. non-Appalachia), nature of injury, cause of injury, and body part injured. Information on job tenure and length of time off work after injury are not required fields and not well populated in the WC FROI system (missing values 14.3 and 61.9%, respectively). The mechanism of missingness for these variables is unknown and these variables were not included in the statistical model. The statistical significance of possible effect modifiers (e.g., age and gender) was evaluated by including their two-way interactions with the SLE sector in the multiple logistic regression model. A backward elimination analysis was used to build a final parsimonious logistic regression model and to estimate the adjusted odds ratio for an injured SLE worker in the Private vs. the Public sector to receive a WC award. Statistical significance was determined with a threshold *p*-value of 0.05. Hosmer-Lemeshow goodness-of-fit test (*p* = 0.80) indicated adequate fit for the final model. Predictive accuracy was measured by c-statistics (0.71) and max-rescaled R^2^ (0.13). SAS^®^ 9.4 software was used for the statistical analysis.

## Results

### Demographic characteristics

The majority of FROIs in both Public and Private SLE were for males (Table 1). Males represented 77% of the Private SLE FROIs and 88% of Public SLE FROIs. There was a greater percentage of young worker FROIs in Private SLE compared to Public SLE. The highest percentage of FROIs in Public SLE was for the 25–44-year-old worker age group (75% Public vs. 42% Private SLE). Regarding job tenure, 36% of Private SLE FROIs were for workers with job tenures of less than 1 year compared to 12% of Public SLE FROIs. Of note, 17% of Private SLE and 14% of Public SLE FROIs had missing data values for job tenure. Regarding occupation listed in SLE FROIs, 91% of Private SLE FROIs was for security guard and Private police occupations, while 96% of Public SLE FROIs was for police officer and detective occupations. When classified by industry, 84% of Private SLE FROIs was in the services industry, primarily Detective, Guard, and Armored Car Services (54%). “Other services” included building maintenance services, combination utility services, individual and family services, legal services, etc. All of the Public SLE FROIs were in the Public administration industry. An increased percentage of Private SLE FROIs were from workers outside Kentucky (12%) compared to Public SLE FROIs (3%).Private SLE FROIs had low percentages from Non-Appalachian and Appalachian residents compared to Public SLE. When location of injury was examined, a higher percentage of Public SLE injuries occurred in Non-Appalachia (81%) compared to the Private SLE injuries (77%).

**Table 1:** Demograhpics of Kentucky Private vs. Public Security and Law Enforcement (SLE) Sector First Reports of Injuries, 2005–2015

Demographic Characteristics	Private SLE Sector Number (%)	Public SLE Sector Number (%)	Chi Square *p*-value
Gender	*n* = 899	*n* = 3478	< 0.001
Male	696 (77%)	3065 (88%)	
Female	202 (23%)	406 (12%)	
Missing^a^	1 (< 1%)	7 (< 1%)	
Age (Years)	*n* = 899	*n* = 3478	< 0.001
Mean	43 (S.E. = 0.495)	37 (S.E. = 0.158)	
≤ 24	98 (11%)	184 (5%)	
25–34	194 (22%)	1315 (38%)	
35–44	179 (20%)	1277 (37%)	
45–54	206 (23%)	522 (15%)	
55+	221 (25%)	179 (5%)	
Missing^a^	1 (< 1%)	1 (< 1%)	
Job Tenure	n = 899	*n* = 3478	< 0.001
< 1 year	327 (36%)	413 (12%)	
≥ 1 year	415 (46%)	2596 (75%)	
Missing^a^	157 (17%)	469 (14%)	
Occupation	*n* = 899	*n* = 3478	< 0.001
Guards & Police Ex. Public Service	808 (91%)	34 (1%)	
Police & Detectives Public Service	73 (8%)	3320 (96%)	
Supervisors of Police & Detectives	< 5 (< 1%)	80 (2%)	
All Others	< 5 (< 1%)	23 (< 1%)	
Missing^a^	13 (1%)	21 (< 1%)	
Industry	*n* = 899	*n* = 3478	
Agriculture, Forestry, and Fishing	28 (3%)	0 (0%)	
Mining & Construction	8 (1%)	0 (0%)	
Manufacturing	28 (3%)	0 (0%)	
Transportation, Communications, Electric, Gas, Sanitary Services	26 (3%)	0 (0%)	
Wholesale Trade	41 (5%)	0 (0%)	
Finance, Insurance, Real Estate	16 (2%)	0 (0%)	
Services	752 (84%)	0 (0%)	
Detective, Guard, and Armored Car Services	485 (54%)	0 (0%)	
Help Supply Service	20 (2%)	0 (0%)	
Security Systems Services	15 (2%)	0 (0%)	
Airports, Flying Fields, and Airport Terminal Services	9 (1%)	0 (0%)	
Other Services	223 (25%)	0 (0%)	
Public Administration	0 (0%)	3478 (100%)	
SLE Worker Residence Region	*n* = 899	*n* = 3478	< 0.001
Appalachia	194 (22%)	955 (27%)	
Non-Appalachia	596 (66%)	2421 (70%)	
Out of State	109 (12%)	102 (3%)	
Location of SLE Worker Injury	*n* = 899	*n* = 3478	< 0.001
Appalachia	166 (18%)	655 (19%)	
Non-Appalachia	689 (77%)	2815 (81%)	
Out of State	35 (4%)	< 10 (< 1%)	
Unknown	9 (1%)	< 5 (< 1%)	

^a^Excluded from statistical analysis

### Injury characteristics

Significant differences were observed in FROI causes of injury between the two SLE sectors (Table 2). A higher percentage of sprains occurred in Public SLE compared to Private SLE (47% vs. 37%, respectively). Falls and slips accounted for 36% of FROIs reported to workers’ compensation by Private SLE personnel, compared to 19% in Public SLE. There were greater proportions of FROIs involving motor vehicle-related injuries, strains, and injuries sustained as a result of a person in the act of a crime in the Public SLE compared to Private SLE.

**Table 2:** Kentucky Private vs. Public Security and Law Enforcement (SLE) Sector Injury Characteristics, 2005–2015

Injury Characteristic	Private SLE sector number (%)	Public SLE sector number (%)	Chi Square *p*-value
Nature of Injury	n = 899	n = 3478	< 0.001
Concussion	< 10 (1%)	36 (1%)	
Contusion	134 (15%)	429 (12%)	
Fracture/dislocation	105 (12%)	314 (9%)	
Gunshot	< 5 (< 1%)	23 (< 1%)	
Laceration/puncture	64 (7%)	276 (8%)	
Sprain/strain	334 (37%)	1634 (47%)	
All Other	252 (28%)	766 (22%)	
Cause of Injury	n = 899	n = 3478	< 0.001
Absorption, ingestion or inhalation	19 (2%)	119 (3%)	
Cut, puncture, or scrape	12 (1%)	86 (2%)	
Fall/slip	325 (36%)	664 (19%)	
Motor vehicle-related	96 (11%)	550 (16%)	
Person in act of crime	38 (4%)	274 (8%)	
Strain	188 (21%)	944 (27%)	
Struck by animal or object	89 (10%)	426 (12%)	
Struck or injured by fellow worker, patient or other person	48 (5%)	120 (4%)	
All other	84 (9%)	295 (9%)	
Body Part Injured	n = 899	*n* = 3478	< 0.001
Head and neck	52 (6%)	158 (5%)	
Face, eyes, mouth, and ears	47 (5%)	133 (4%)	
Upper extremity	186 (21%)	1002 (29%)	
Back	105 (12%)	265 (8%)	
Chest and abdomen, including groin	72 (8%)	280 (8%)	
Pelvis and upper leg	23 (3%)	91 (3%)	
Ankle and foot	81 (9%)	291 (8%)	
Knee and lower leg	169 (19%)	663 (19%)	
Multiple parts, whole body, or body systems	151 (17%)	543 (16%)	
No physical injury	7 (< 1%)	40 (1%)	
Insufficient information	6 (< 1%)	12 (< 1%)	
Length of Time Off After Injury	*n* = 899	*n* = 3478	0.001
No lost time	23 (3%)	85 (2%)	
< 14 days	207 (23%)	1033 (30%)	
≥ 14 days and < 30 days	32 (4%)	82 (2%)	
≥ 30 days	52 (6%)	153 (4%)	
Missing values	585 (65%)	2125 (61%)	

In regards to the location of injuries on the body, there were 8% more upper extremity injuries listed in Public SLE FROIs, while there were 4% more back injuries in Private SLE FROIs. There was a higher percentage of Private SLE FROIs that resulted in two or more weeks off work following the injury compared to Public SLE FROIs (9% of Private SLE FROIs vs. 7% of Public SLE FROIs); 30% of Public SLE FROIs resulted in less than 14 days off work compared to 23% of Private SLE FROIs. There was a large proportion of FROIs with missing values for length of time off work, with 65% missing in Private SLE FROIs, and 61% missing in Public SLE FROIs.

### Disposition status

The large majority of FROIs in both SLE sectors received no awarded benefits (80% of Private SLE and 84% of Public SLE, respectively) (Table 3). Awarded benefits were issued as a lump sum or agreement on the FROI and via an agreement or award reached with an ALJ. A higher proportion of FROIs was compensated via all methods in Private SLE (19%) compared to Public SLE (14%). FROIs with a disposition categorized as “other” were those FROIs awaiting a final decision for various reasons.

**Table 3:** Disposition Status of Kentucky Private vs. Public Security and Law Enforcement (SLE) Sector First Reports of Injuries, 2005–2015

First report of injury Disposition	Private SLE sector number (%)	Public SLE sector number (%)	Chi Square *p*-value
Disposition	*n* = 899	*n* = 3478	< 0.001
None	722 (80%)	2920 (84%)	
Lump sum agreement on first report	80 (9%)	270 (8%)	
Agreement approved by administrative law judge	67 (8%)	146 (4%)	
Agreement approved on first report	< 5 (< 1%)	58 (2%)	
Award (by administrative law judge)	14 (2%)	21 (< 1%)	
Other	< 15 (1%)	63 (2%)	
FROI Resulted in Workers’ Compensation Award			
Disposition	*n* = 899	*n* = 3478	< 0.001
No (no award)	724 (81%)	2935 (84%)	0.004
Yes	168 (19%)	501 (14%)	
Under Review	7 (1%)	42 (1%)	

### Multiple logistic regression

As shown in Table 4, SLE sector, age, nature of injury, cause of injury, and body part injured were all selected as covariates in the final model. The estimated adjusted odds that a FROI would result in awarded benefits was higher (OR = 1.354; 95% CI[1.086,1.688]) for Private SLE workers compared to Public SLE workers when adjusting for age, nature of injury, cause of injury, and injured body part. Middle-aged SLE employees (45–54 year olds) had the highest adjusted odds (OR = 3.254; 95% CI [1.964-,5.390]) of an awarded benefit compared to the youngest age group after adjusting for SLE sector, nature of injury, cause of injury, and body part injured. Gunshot wounds (OR = 3.771; 95% CI [1.340, 10.612]) and fractures/dislocations (OR = 1.704, 95% CI [1.215, 2.389]) resulted in increased adjusted odds of awarded benefits compared to “all other” nature of injuries, while contusions (OR = 0.531; 95% CI [0.327-, 0.757]) and lacerations/punctures (OR = 0.218; 95% CI]0.108, 0.441]) resulted in decreased adjusted odds of awarded benefits, compared to “all other” injuries. Motor vehicle injuries FROIs had the highest adjusted odds of an awarded benefit compared to “all other” causes of injury (OR = 5.274; 95% CI [3.156-, 8.813]). Fall and slip, strain, and person in the act of crime FROIs also had higher adjusted odds of resulting in awarded benefits compared to “all other” injuries.

**Table 4:** Multiple Logistic Regression Predicting the Probability that a Kentucky Security and Law Enforcement (SLE) First Report of Injury will Result in Awarded Benefits^a^

Variable	Adj. Odds Ratio	Confidence Interval
SLE Sector		
Public	Reference	
Private	1.354	(1.086, 1.688)
Age (Years)		
≤ 24	Reference	
25–34	1.911	(1.165, 3.137)
35–44	2.663	(1.628, 4.357)
45–54	3.254	(1.964, 5.390)
55+	2.941	(1.721, 5.026)
Nature of injury		
All Other	Reference	
Concussion	1.092	(0.465, 2.561)
Contusion	0.531	(0.327, 0.757)
Fracture/dislocation	1.704	(1.215, 2.389)
Gunshot	3.771	(1.340, 10.612)
Laceration/puncture	0.218	(0.108, 0.441)
Sprain/strain	0.956	(0.732, 1.249)
Cause of injury		
All other	Reference	
Absorption, ingestion or inhalation	0.254	(0.058, 1.109)
Cut, puncture, or scrape	2.293	(0.742, 7.090)
Fall/slip	3.022	(1.816, 5.027)
Motor vehicle-related	5.274	(3.156, 8.813)
Person in act of crime	3.025	(1.716, 5.333)
Strain	3.235	(1.933, 5.413)
Struck by animal or object	1.799	(1.017, 3.180)
Struck or injured by fellow worker, patient or other person	0.976	(0.426, 2.235)
Body part injured		
Ankle and foot	Reference	
Back	2.372	(1.515, 3.715)
Chest, abdomen, and groin	0.786	(0.438, 1.408)
Face, eyes, mouth and ears	1.322	(0.611, 2.861)
Head and neck	1.925	(1.075, 3.447)
Knee and lower leg	3.078	(2.062, 4.592)
Multiple parts or body systems	2.274	(1.452, 3.561)
Pelvis and upper leg	1.748	(0.881, 3.467)
Upper extremity	2.471	(1.663, 3.674)
Missing	0.000	–

^a^Award outcome only included first reports of injuries where a decision was reached. Cases under review (*n* = 49) were excluded

## Discussion

This study identified that Private SLE FROIs had higher adjusted odds of resulting in awarded benefits compared to Public SLE FROIs that may represent more equitable workers’ compensation and lengthier disability for fall injuries and back injuries in Private SLE. The higher percentage of awarded benefits in Private SLE based on an agreement with an administrative law judge could be due to a number of factors, including primary industry of employment for Private SLE workers. Twenty-five percent of Private SLE FROIs were in the other services industries. The process may be more difficult to receive temporary disability, and permanent disability payments for temporary Private SLE employees in the other services industries compared to Public SLE where the primary employement industry is public administration that may be more amenable to parties’ agreement on adequate compensation for SLE injuries. A second factor for the increased percentage of awarded benefits based on resolution of claims by an ALJ may be due to injury types identified in the Private SLE. There was a higher percentage of fall injuries that resulted in back injuries in Private SLE compared to Public SLE. Back injuries typically result in higher median costs per workers’ claim, and total costs for both genders, as well as longer compensation benefit duration (Lederer and Rivard 2014). Since back claims are so costly, ALJ adjudication may occur more often to resolve compensation monetary award disputes.

While there are a number of publications on law enforcement-related injuries, few have used workers’ compensation data, and none, to the knowledge of the authors, have examined differences between Public and Private SLE. An occupational injury surveillance study compared correctional officer, municipal police, sheriff’s officer, and state police workers’ claims by occupation, but not by industry sector (Holloway-Beth et al. 2016). The authors reported similar mean ages, gender, and injury types (motor vehicle-related, fall, and overexertion injuries, in addition to assaults) to the results of this study that help contribute to the generalizability of this study to larger populations of law enforcement occupations. Kentucky’s workers’ compensation system only requires an employee to miss one day of work that is useful in identifying total law enforcement injuries compared to other state workers’ compensation systems with more stringent injury reporting regulations (Kentucky Regulatory Statute 342.038) (e.g., seven days) where only the most severe injuries may be reported to workers’ compensation.

In this study, a greater proportion of Private SLE FROIs were for employees with short job tenures; there were also higher percentages for younger and older employees compared to Public SLE FROIs. These differences between Private vs. Public SLE could be due to differences in new and refresher employee safety training between Private and Public SLE, or due to stage of career employment between Private vs. Public SLE. There could also be differences in injury reporting between Private and Public SLE; FROI reporting rates could not be determined since Private and Public SLE employment crosses multiple industries and occupations.

A limitation to this study was a change in the occupation data field in March 2011. Prior to 2011, FROIs utilized standardized occupation codes to report the injured worker’s occupation. When upgrading to the 3.0 release of claims standards set forth by the International Association of Industrial Accident Boards and Commissions (IAIABC), the occupation data field transitioned from a standard text code to a free form text field. This upgrade in the reporting system could have resulted in undercounting of FROIs by occupation, including SLE FROIs. The IAIABC should reconsider its decision on the use of free form text to identify occupation. To better target injury prevention efforts in Private and Public SLE, as well as in other industries in states using IAIABC data coding schema, accurate numbers and type of injuries by standard occupational classifications are necessary; adding a drop down menu of standard occupational codes to workers’ compensation first report of injury forms may be useful.

Differing work organization and workers’ compensation coverage between Private and Public SLE also could have contributed to possible underreporting. Security guards can be hired as independent contractors, which may not be covered by workers’ compensation, and would exclude them from this data set. Temporary workers may be covered under the temporary employment agency workers’ compensation coverage; volunteer police workers are covered by workers’ compensation in Kentucky.

In addition, a limitation of workers’ compensation data is its specificity in identifying cause and severity of injury. FROIs do not include a diagnosis from a medical professional, utilize a text field for the location of the injury, and do not include detailed information regarding the severity of the injury. Causes of injury are general in nature regarding the mechanism of injury (e.g.., ‘person in act of crime’).

## Conclusion

The results of this study suggest that Private SLE is more likely to have FROIs resulting in awarded benefits compared to those employed in Public SLE. Compared to Public SLE workers, there was a higher percentage of fall and back injuries among Private SLE employees, with higher percentages of FROIs in younger Private SLE workers < 25 years of age, mature Private SLE workers 45 years of age and older, and Private SLE workers with job tenures under one year.

These findings may be useful in developing targeted worker safety trainings for Private SLE on fall prevention and the prevention of back injuries, and for Public SLE on the prevention of fall injuries and motor vehicle safety. In addition, improvement in the completeness of workers’ compensation data through addition of data fields in first reports of injury and in IAIABC coding schema is needed to improve identification of occupation, and to better characterize SLE and other occupation-specific injuries.

## References

[CR1] ASIS International. Security Glossary- P. “Private Security”. 2009. https://admin.asisonline.org/Membership/Library/Security-Glossary/Pages/Security-Glossary-P.aspx. Accessed 8 Dec 2017.

[CR2] Banks D, Hendrix J, Hickman M, Kyckelhahn T. National Sources of law enforcement employment data. Bureau of Justice Statistics. NCJ 249681. Published April 2016. Accessed 8 Dec 2017.

[CR3] Bunn TL, Slavova S, Tang M (2011). Injuries among solid waste collectors in the private versus public sectors. Waste Manag Res.

[CR4] Bureau of Labor Statistics. U.S. Department of Labor. Occupational employment statistics: security guards (code 339032). Published 31 Mar 2017a. http://www.bls.gov/oes/current/oes339032.htm. Accessed 8 Dec 2017.

[CR5] Bureau of Labor Statistics. U.S. Department of Labor. Occupational employment statistics: detectives and investigators (code 333021). Published 31 Mar 2017b. http://www.bls.gov/oes/current/oes339021.htm. Accessed 8 Dec 2017.

[CR6] Bureau of Labor Statistics. U.S. Department of Labor. Occupational employment statistics: Kentucky, may 2016. Published 31 Mar 2017c. http://www.bls.gov/oes/current/oes_ky.htm#33-0000. Accessed 8 Dec 2017.

[CR7] Bureau of Labor Statistics. Occupational injuries and illness and fatal injuries, 2016. Published 31 Mar 2017d. http://data.bls.gov/gqt/InitialPage. Accessed 8 Dec 2017.

[CR8] Fan ZJ, Foley MP, Rauser E, Bonauto DK, Silverstein BA (2013). Effects of residential location and work-commuting on long-term work disability. J Occup Rehabil.

[CR9] Holloway-Beth A, Forst L, Freels S, Brandt-Rauf S, Friedman L (2016). Occupational injury surveillance among law enforcement officers using workers’ compensation data, Illinois. 1980 to 2008. Journal Occup Environ Med.

[CR10] Kentucky Revised Statutes (1980). Kentucky regulatory statute 342.038.

[CR11] Lederer V, Rivard M (2014). Compensation benefits in a population-based cohort of men and women on long-term disability after musculoskeletal injuries: costs, course, predictors. Occup Environ Med.

[CR12] Sparrow, MK. Managing the boundary between Public and Private policing. Executive Session on Policing and Public Safety. New Perspectives in Policing. September 2014. Harvard Kennedy School, and National Institute of Justice.

[CR13] Strom K, Berzofsky M, Shook-Sa B, Barrick K, Daye C, Horstmann N, Kinsey S. The private security industry: a review of the definitions, available data sources, and paths moving forward. 2010. RTI International: Bureau of Justice Statistics. https://www.ncjrs.gov/pdffiles1/bjs/grants/232781.pdf. Accessed 9 Dec 2017.

[CR14] Tiesman HM, Swedler DI, Konda S, Pollack KM (2013). Fatal occupational injuries among U.S. law enforcement officers: a comparison of national surveillance systems. Amer J Ind Med.

[CR15] Young AE, Wasiak R, Webster BS, Shayne RG (2008). Urban-rural differences in work disability after an occupational injury. Scand J Work Environ Health.

